# Protein Expression Landscape Defines the Formation Potential of Mouse Blastoids From EPSCs

**DOI:** 10.3389/fcell.2022.840492

**Published:** 2022-02-08

**Authors:** Zheying Min, Ke Zhong, Yuxin Luo, Yong Fan, Yang Yu

**Affiliations:** ^1^ Department of Obstetrics and Gynecology, Key Laboratory for Major Obstetric Diseases of Guangdong Province, Key Laboratory of Reproduction and Genetics of Guangdong Higher Education Institutes, The Third Affiliated Hospital of Guangzhou Medical University, Guangzhou, China; ^2^ Beijing Key Laboratory of Reproductive Endocrinology and Assisted Reproductive Technology and Key Laboratory of Assisted Reproduction, Ministry of Education, Center of Reproductive Medicine, Department of Obstetrics and Gynecology, Peking University Third Hospital, Beijing, China; ^3^ Stem Cell Research Center, Peking University Third Hospital, Beijing, China

**Keywords:** blastoids, mEPSCs, proteome, glucose metabolism, phosphorylation, post translation modifications

## Abstract

Preimplantation embryo development is a precisely regulated process organized by maternally inherited and newly synthesized proteins. Recently, some studies have reported that blastocyst-like structures, named blastoids, can be generated from mouse ESCs (embryonic stem cells) or EPSCs (extended pluripotent stem cells). In this study, to explore the dynamic expression characteristics of proteins and their PTMs in mouse EPS blastoids, we revealed the protein expression profile of EPS blastoids and metabolite characteristics by TMT-based quantitative mass spectrometry (MS) strategy. Furthermore, the protein phosphorylation sites were identified to show the phosphoproteomic analysis in blastoids compared with mouse early embryos. Above all, our study revealed the protein expression profile of EPS blastoids compared with mouse embryos during preimplantation development and indicated that glucose metabolism is key to blastoid formation.

## Introduction

Preimplantation embryo development is a precisely regulated process organized by maternally inherited and newly synthesized proteins. After fertilization, the embryo is controlled by maternal RNAs and proteins before ZGA (zygotic gene activation) (Xue et al., 2013). The blastomeres then compact, polarize and generate the first two lineage segregations of the inner cell mass (ICM) and the trophectoderm (TE). There are many questions to be answered regarding this process. Recently, some studies have reported that blastocyst-like structures, named blastoids, can be generated from mouse ESCs (embryonic stem cells) or EPSCs (extended pluripotent stem cells), which can partially mimic early mouse embryo development *in vitro* (Rivron et al., 2018; Li et al., 2019; Sozen et al., 2019). This new model of early embryo development needs to be better understood because of differences in developmental efficiency, gene expression and biofunction. Proteins mediate most biological processes, and the biological function of a protein requires posttranslational modifications (PTMs) (Schwanhausser et al., 2013; Snider and Omary, 2014). The proteome landscape can provide direct access to the molecular details of early embryos. Understanding the dynamic changes in the mouse EPS blastoid proteome and PTMs provides insight into the mechanism of blastocyst-like structure generation and natural mouse embryogenesis.

## Materials and Methods

### EPS Blastoids Generation

EPSCs were cultured on fresh mitomycin C-treated MEFs in N2B27 basal medium supplemented with 10 ng/ml LIF (R&D Systems, 7,734), 3 mM CHIR99021 (Tocris, 4,423), 2 mM (S)-(+)-dimethylene maleate (Tocris, 1,425), and 2 mM minocycline hydrochloride (Selleckchem, S4226) (hereinafter referred to as N2B27-LCDM). The N2B27-LCDM medium was changed every day. N2B27 basal medium was prepared as follows: 240 ml DMEM/F12 (Thermo Fisher Scientific, 11330-032), 240 ml neurobasal (Thermo Fisher Scientific, 21103-049), 2.5 ml N2 supplement (Thermo Fisher Scientific, 17502-048), 5 ml B27 supplement (Thermo Fisher Scientific, 12587-010), 1% nonessential amino acids (Thermo Fisher Scientific, 11140-050), 1% L-glutaMAX (Thermo Fisher Scientific, 35050-061), 0.1 mM *β*-mercaptoethanol (Thermo Fisher Scientific, 21985-023), 1% penicillin/streptomycin (Thermo Fisher Scientific, 15140-122), and 5% knockout serum replacement (KSR, Thermo Fisher Scientific, A3181502). EPS colonies were dissociated into single cells by TrypLE Express Enzyme (Thermo Fisher Scientific, 12604021), and the MEFs were transferred to a 0.2% gelatin-coated plate at 37°C for 30 min. AggreWell 400 (STEMCELL Technologies, 34415) was prepared following the manufacturer’s instructions. EPS blastoid basal medium was composed of 25% TSC basal medium (see above), 25% N2B27 basal medium (see above), and 50% KSOM (see above). Approximately 6,000 cells (5 cells per microwell for 1,200 microwells) were resuspended in EPS blastoids basal medium supplemented with 2 mM ROCK inhibitor Y-27632 (Selleckchem, S1049), 12.5 ng/ml rhFGF4 (R&D, 235-F4), 0.5 mg/ml heparin (Sigma, H3149), 3 mM CHIR99021 (Tocris, 4,423), and 0.5 mM A83-01 (Selleckchem, S7692) and seeded into one well of a 24-well AggreWell plate. The blastoid culturing conditions were as follows: 37°C, 20% O_2_, 5% CO_2_ and saturated humidity. The day of cell seeding was counted as Day 0 of the process. The medium was removed 24 h later (Day 1) and replaced with fresh medium supplemented with 5 ng/ml BMP4 (R&D Systems, 314-BP-010) and without Y-27632. EPS aggregates and blastoids were manually picked up using a mouth pipette (homemade) or analysis or downstream experiments. To test the effect of 2-DG on EPS blastoid induction, chemicals were added to the medium at Day 1 for 72 h.

### Immunofluorescence Staining

The samples were fixed with 4% paraformaldehyde in phosphate buffered saline (PBS) for 20 min at room temperature, washed three times with PBS, and permeabilized with 0.2% Triton X-100 in PBS for 15–30 min. After blocking with 5% BSA in PBS for 1 h at room temperature, samples were then incubated with primary antibody diluted in blocking buffer overnight at 4°C. After primary antibody incubation, the samples were washed three times with PBS containing 0.1% Tween 20. Samples were washed three times with PBS containing 0.1% Tween 20 and incubated with fluorescence-conjugated secondary antibodies diluted in blocking buffer at temperature for 2 h. Nuclei were stained with Hoechst 33342 (Sigma, 94403) at 1 μg/ml. Zeiss LSM 710 was used for imaging. Images were processed by ZEN (Zeiss) and Fiji (ImageJ, V2.0.0) software. The primary antibodies and dilutions were as follows: mouse anti-OCT4 (Santa Cruz, sc5279, polyclonal, 1:200), anti-CDX2 (Abcam, ab76541, 1:200), and rabbit anti-YAP (Cell Signaling Technology, 14074, monoclonal, 1:200). The secondary antibodies were Alexa Fluor 488 goat anti-rabbit IgG (H + L) (Thermo Fisher Scientific, A-11008), Alexa Fluor 555 goat anti-mouse IgG (H + L) (Cell Signaling Technology, 4409S), and Alexa Fluor 647 goat anti-rabbit IgG (H + L) (Abcam, ab150083, GR3269213).

### Protein Extraction, Trypsin Digestion and TMT Labeling

The EPS blastoids were collected at Day 3 and Day 5. The samples of the two groups were mixed separately, and quantitative phosphorylated proteomics analysis was performed. There were three technical replicates. The sample was sonicated three times on ice using a high-intensity ultrasonic processor (Scientz) in lysis buffer (8 M urea, 1% protease Inhibitor Cocktail). The remaining debris was removed by centrifugation at 12,000 g at 4°C for 10 min. Finally, the supernatant was collected, and the protein concentration was determined with a BCA kit according to the manufacturer’s instructions.

For digestion, the protein solution was reduced with 5 mM dithiothreitol for 30 min at 56°C and alkylated with 11 mM iodoacetamide for 15 min at room temperature in darkness. The protein sample was then diluted by adding 100 mM NH_4_HCO_3_ to a urea concentration less than 2 M. Finally, trypsin was added at a 1:50 trypsin-to-protein mass ratio for the first digestion overnight and a 1:100 trypsin-to-protein mass ratio for a second 4 h digestion.

After trypsin digestion, the peptide was desalted by a Strata X C18 SPE column (Phenomenex) and vacuum-dried. Peptide was reconstituted in 0.5 M TEAB and processed according to the manufacturer’s protocol for the TMT kit. Briefly, one unit of TMT reagent was thawed and reconstituted in acetonitrile. The peptide mixtures were then incubated for 2 h at room temperature and pooled, desalted and dried by vacuum centrifugation. The digested peptides from EPS blastoids at Day 3 and Day 5 with three biological replicates were labeled with TMT^6^-126, TMT^6^-127, TMT^6^-128, TMT^6^-129, TMT^6^-130, and TMT^6^-131 Labeling Reagent (Thermo Fisher Scientific), respectively, following the manufacturer’s protocol.

### Phosphorylated Peptide Enrichment

To enrich phosphorylation-modified peptides, most of labeled peptide was dissolved in the enrichment buffer solution (50% acetonitrile/0.5% acetic acid), and the supernatant was transferred to the pre-washed IMAC material, which was placed on a rotating shaker and gently shaken for incubation. After incubation, the material was washed three times with the buffer solution (50% acetonitrile/0.5% acetic acid and 30% acetonitrile/0.1% trifluoroacetic acid) successively. Finally, the phosphopeptides were eluted from the materials with 10% ammonia water, and the eluted fractions were combined and vacuum-dried (Riley and Coon, 2016).

### Reversed-Phase-High Performance Liquid Chromatography Fraction

The tryptic peptides were fractionated into fractions by high pH reverse-phase HPLC using Thermo Betasil C18 column (5 μm particles, 10 mm ID, 250 mm length). Briefly, peptides were first separated with a gradient of 8–32% acetonitrile (pH 9.0) over 60 min into 60 fractions. Then, the peptides were combined into six fractions and dried by vacuum centrifuging.

### Liquid Chromatography With Tandem Mass Spectrometry Analysis

For LC–MS/MS analysis, the resulting peptides were desalted with C18 ZipTips (Millipore) according to the manufacturer’s instructions. To ensure the high confidence identification of the results, the identification data was filtered with the criterion of localization probability >0.75.

These peptides were dissolved in 0.1% formic acid (solvent A) and directly loaded onto a homemade reversed-phase analytical column (15 cm length, 75 μm i. d.). The gradient was comprised of a solvent B (0.1% formic acid in 98% acetonitrile) with an increase from 6% to 23% over 26 min, then an increase from 23% to 35% over 8 min, a climb to 80% over 3 min, and a hold at 80% for the last 3 min, all at a constant flow rate of 400 nL/min on an EASY-nLC 1000 UPLC system.

The peptides were subjected to an NSI source followed by tandem mass spectrometry (MS/MS) in Q ExactiveTM Plus (Thermo) coupled online to UPLC. The electrospray voltage applied was 2.0 kV. The m/z scan range was 350–1800 for a full scan, and intact peptides were detected in the Orbitrap at a resolution of 70,000. Peptides were then selected for MS/MS using the NCE setting of 28, and the fragments were detected in the Orbitrap at a resolution of 17,500. A data-dependent procedure that alternated between one MS scan followed by 20 MS/MS scans with a 15.0 s dynamic exclusion was used. The automatic gain control (AGC) was set at 5E4. Fixed first mass was set as 100 m/z.

### Mass Spectrometry-Based Global Analysis of Phosphorylation

To identify protein phosphorylation, we used the SEQUEST algorithm in the Proteome Discoverer software suite (Thermo Fisher Scientific). The search parameters included a differential modification on serine, threonine, and tyrosine residues of 79.9663 amu, indicating the addition of phosphorous group(s). Proteome Discoverer further calculated the quantitative information of the TMT-tagged reporter ions at the modified peptide level. And the TMT quantified significance difference threshold was 1.3 times. For phosphorylation, quantitative information and localized phosphorylation sites were assembled to derive quantified phosphorylation sites.

### Metabolomics

To detect as many metabolites as possible, untargeted metabolomics profiling was performed on the XploreMET platform (Metabo-Profile, Shanghai, China). The sample preparation procedures were performed according to previously published methods with modifications (Zhen et al., 2019).

### Statistical Analysis

Data are presented as the mean values ±SEM. Comparisons between the two groups were determined by two-tailed Student’s t test. Statistical analyses were performed with GraphPad Prism software for individual analysis, and statistical significance is shown as not significant (NS), *p < .05, **p < .01.

### Motif Analysis of Phosphorylation Sites

Software MoMo and Motif-X algorithms were used to analyze the motif characteristics of the phosphorylation sites. Peptide sequences composed of six amino acids in the upstream and downstream of all identified phosphorylation sites were analyzed. Analysis and comparison background were peptide sequences of six amino acids upstream and downstream of all potential phosphorylation sites in species. When the number of peptides in a characteristic sequence form was greater than 20 and the statistical test *p* value was less than .000001, the characteristic sequence form was considered as a motif of the phosphorylation peptide.

## Results

### Dynamic Expression Characteristics of Proteins and PTMs in Mouse EPS Blastoids

**FIGURE 1:**
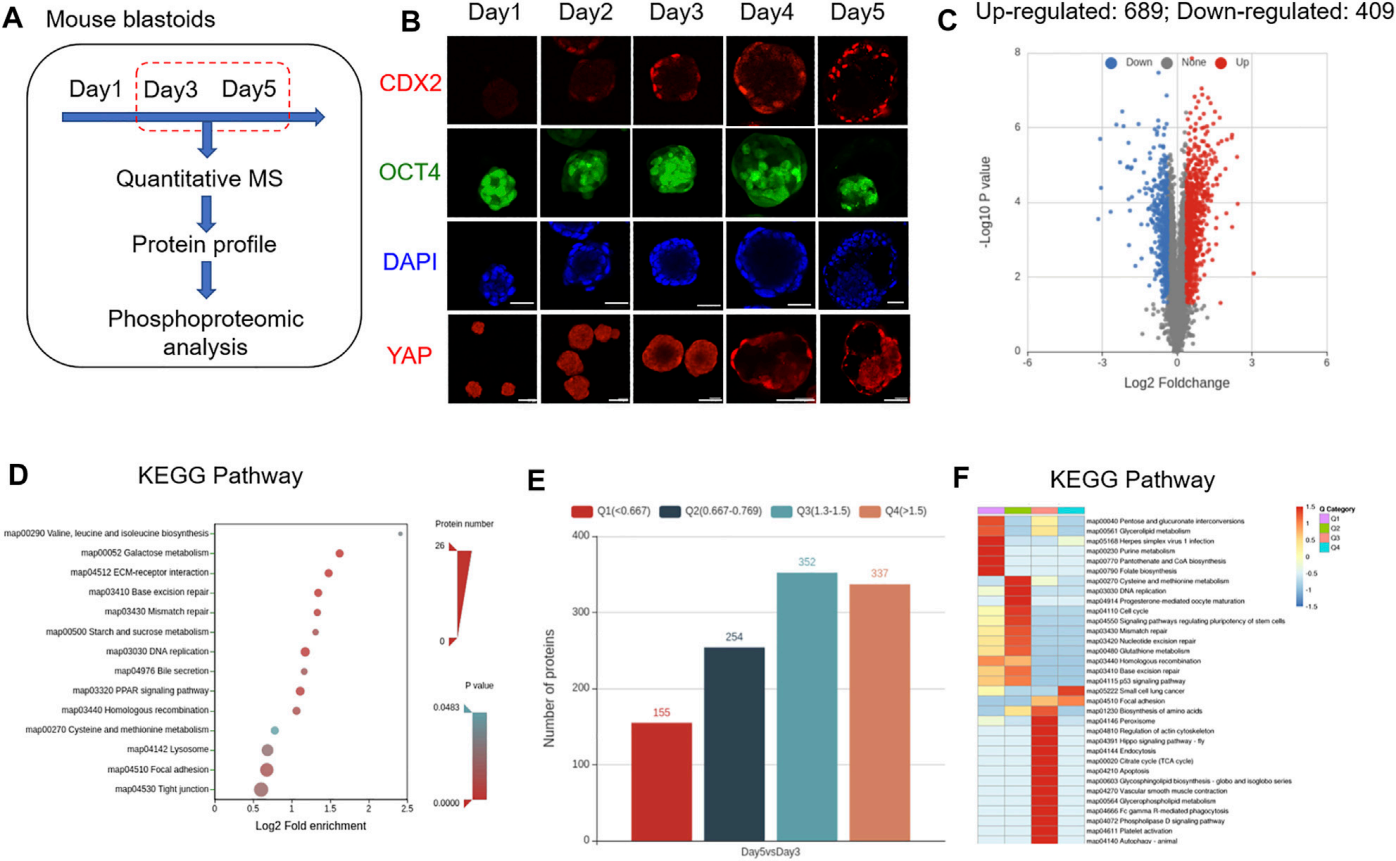
Protein expression characteristics of mouse blastoids. **(A)** Diagram of blastoid collection and LC–MS/MS analysis. **(B)** Immunofluorescence staining of EPS aggregates on the indicated day for CDX2, OCT4 and YAP expression. Scale bar = 50 μm. **(C)** Volcano plots showing differentially expressed proteins between blastoids at Day 5 and Day 3. **(D)** KEGG pathway analysis of the biological processes of differentially expressed proteins. **(E)** The number of up- and downregulated proteins in blastoids at Day 5 compared with Day 3. The differentially expressed proteins were divided into four quantified groups based on their differential expression multiples: Q1 (0 < ratio ≤0.667), Q2 (0.667 < ratio ≤0.769), Q3 (1.3 < ratio ≤1.5), and Q4 (ratio >1.5). **(F)** KEGG pathway enrichment of quantitative differentially expressed proteins in each Q group.

To explore the dynamic expression characteristics of proteins and their PTMs in mouse EPS blastoids, a tandem mass tag TMT-based quantitative mass spectrometry (MS) strategy was used. Based on TMT-based quantitative MS, we analyzed the protein expression profiles of blastoids at Day 3 and Day 5, which are the key stages for embryo polarization and lineage specification (Figure 1A). For each stage, 30,000 blastoids were selected, and the experiment was performed in three biological replicates. During 5 days of blastoid development, dynamic changes in the TE marker CDX2 and EPI marker OCT4 were observed (Figure 1B). We identified approximately 6,324 proteins from 30,000 EPS blastoids each (∼5 × 10^6^ cells) at Day 3 and Day 5. All peptides identified were of high mass accuracy and had good repeatability (Supplementary Figures S1A,B). The distribution of protein sequence coverage was very similar among the three replicates (Supplementary Figure S1C). When comparing blastoids between Day 5 and Day 3, the upregulated proteins were 689, and the downregulated proteins were 409 (Figure 1C). KEGG pathway analysis of differentially expressed proteins in blastoids revealed that these proteins were enriched in amino biosynthesis and galactose/sucrose metabolism (Figure 1D). These differentially expressed proteins were divided into four quantified groups based on their differential expression multiples: Q1 (0 < ratio ≤0.667), Q2 (0.667 < ratio ≤0.769), Q3 (1.3 < ratio ≤1.5), and Q4 (ratio >1.5) (Figure 1E). For the four quantified groups, Q1 and Q2 downregulated proteins were enriched in glycerolipid, purine and amino acid metabolism. Q3 and Q4 upregulated proteins were enriched in the Hippo signaling pathway, TCA cycle and apoptosis (Figure 1F).

### Glucose Metabolism Influenced the Formation of Mouse Blastoids and Embryogenesis

**FIGURE 2:**
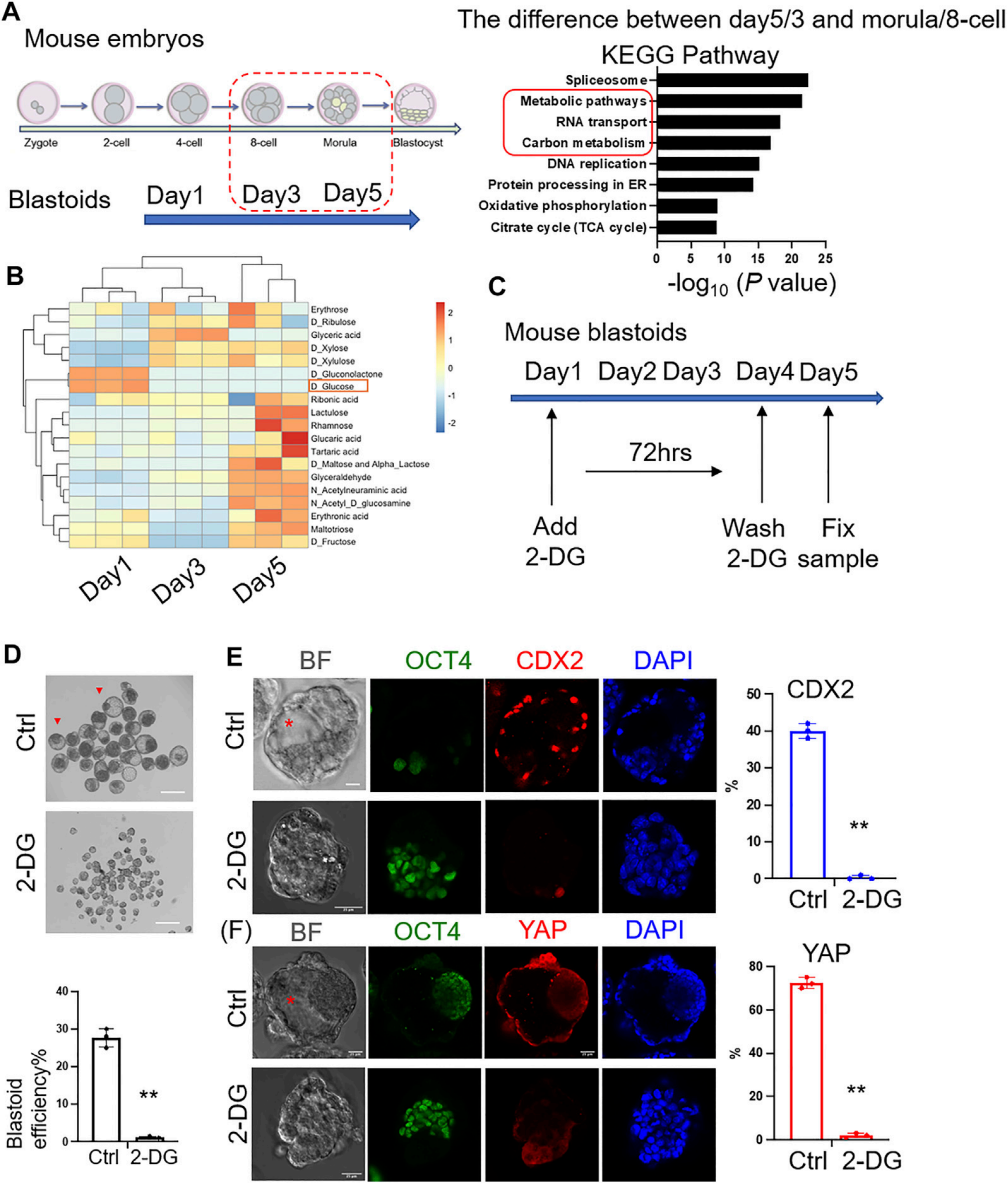
Importance of glucose metabolism to the formation of mouse blastoids. **(A)** KEGG analysis of differentially expressed proteins between blastoids and mouse embryos at the indicated stages. **(B)** Metabolites of blastoids and cultured medium of blastoids at Days 1, 3 and 5. **(C)** EPS aggregates cultured in control and 2-DG medium. 2-DG was added to the medium on Day 1 and washed off on Day 4 (∼72 h). **(D)** Phase-contrast images of EPS aggregates after 5 days of blastoid culture medium supplemented with vehicle (top) and 2-DG (down). The quantification of blastoid formation efficiency with the indicated treatment was performed. Scale bar = 150 μm **, *p* < .01. **(E)** Immunofluorescence staining of EPS aggregates of the control and 2-DG-treated groups at Day 5 for CDX2 expression. Scale bar = 25 μm **, *p* < .01. Red asterisk indicates cavity. **(F)** Immunofluorescence staining of EPS aggregates of the control and 2-DG-treated groups at Day 5 for YAP expression. Scale bar = 25 μm **, *p* < .01. Red asterisk indicates cavity.

To compare the similarities of protein expression between mouse EPS blastoids and preimplantation mouse embryos (Gao et al., 2017), unsupervised hierarchical clustering was analyzed and indicated that the protein expression profiles of blastoids at Days 3 and 5 were similar to the 8-cell to morula stage mouse embryos (Supplementary Figures S2A,B). The overlapping number of differentially expressed proteins between Day 5/Day 3 blastoids and morula/8-cell stage embryos was 437 (Supplementary Figure S2C). We next found that the differentially expressed proteins between blastoids and mouse embryos were enriched in metabolic pathways and carbon metabolism (Figure 2A). Then, the metabolite analysis of blastoids at Day 1 to Day 5 showed that the content of glucose was decreased during blastoid culture (Figure 2B). Some research reported that glucose signaling controls TE cell fate and is necessary for TE marker activation and specification in mouse early embryos. Glucose is transported to the cytoplasm of embryos from the external environment and controls the nuclear translocation of YAP through the hexosamine biosynthetic pathway (HBP). Then, it mediates mTOR pathway activation to control TFAP2C translation through pentose phosphate pathway (PPP). The complex formed by nuclear-localized YAP and TFAP2C activates CDX2 expression for TE specification (Chi et al., 2020; Zhu and Zernicka-Goetz, 2020). Therefore, based on the decreased glucose and the above information, we hypothesized that the absorption of glucose was necessary for blastoid formation. To explore the importance of glucose metabolism in mouse embryo development, we sought to test the role of glucose in early blastoid formation. Then, 2-DG (2-deoxy-D-glucose), an analog of glucose, was added to the culture medium to inhibit glucose utilization (Figure 2C). As expected, the efficiency of cavity formation was decreased significantly in the 2-DG-treated group compared with the control group (Figure 2D). The expression percentage of the TE marker CDX2 and TE nuclear localized marker YAP was decreased significantly compared with that of the control group (Figures 2E,F). Our data therefore provide clues on the key role of glucose metabolism in blastoid formation as well as mouse embryogenesis.

### Characteristics and Regulation of Protein Phosphorylation in Mouse Blastoids and Blastocysts

Signal transduction through protein phosphorylation may play an important role during early embryo development; therefore, we combined high-accuracy and high-resolution liquid chromatography-tandem mass spectrometry (LC-MS/MS) to profile the phosphorylation events for serine, threonine, and tyrosine phosphorylation of enriched modified peptides. This analysis identified 7,852 phosphopeptides from 2,935 proteins, indicating widespread activation of phosphorylation in early embryos. When statistically significant difference existed (*p* value was below .05), the change of differential modification quantity over 1.3 was regarded as significant up-regulation, and that less than 1/1.3 was regarded as significant down-regulation. Therefore, the number of hyperphosphorylated sites was 1,054, and the number of hypophosphorylated sites was 883 (Supplementary Figure S3A). All phosphopeptides identified were of high mass accuracy and had good repeatability (Supplementary Figures S1D,E). The overlapping number of differentially phosphorylated proteins between Day 5/Day 3 blastoids and morula/8-cell stage embryos was 447 (Supplementary Figure S2D). KEGG pathway and Gene ontology (GO) analysis of differentially phosphorylated proteins in blastoids at Day 3 and Day 5 revealed that these differential proteins were enriched in RNA splicing and mRNA metabolic processes (Supplementary Figures S3B,C). The distribution of serine, threonine, and tyrosine phosphorylation sites were shown in the Supplementary Figure S3D, serine phosphorylation sites accounted for more than 90%, while tyrosine phosphorylation sites were less than 1%. For four of the quantified phosphorylated protein groups, Q1 and Q2 hypophosphorylated proteins were enriched in the spliceosome, Hippo signaling pathway and amino acid metabolism. Q3 and Q4 hyperphosphorylated proteins were enriched in glycolysis/gluconeogenesis and apoptosis (Supplementary Figure S3E,F). These results were similar to differentially expressed proteins. Furthermore, Hippo Signaling Pathway plays an essential role in the specification of ICM and TE in the early embryo development (Yagi et al., 2007; Nishioka et al., 2009; Kaneko and DePamphilis, 2013; Rayon et al., 2014; Posfai et al., 2017). In the outer cells, the Hippo pathway is quiescent, and the unphosphorylated YAP enters the nucleus and binds to Tead4 to form a complex that activates the expression of TE-specific genes, promoting TE differentiation. In the inner cells, the Hippo pathway is activated and YAP is phosphorylated. Phosphorylated YAP is retained in the cytoplasm and subsequently degraded, preventing the expression of TE-specific genes. Instead, abundant expression of pluripotent transcription factors promotes ICM differentiation. The formation of TE and ICM analogs is also a key event for the successful generation of mouse blastoids. We therefore explored the phosphorylation and cellular localization of YAP in mouse blastoids. Immunofluorescence staining of EPS aggregates showed that YAP was predominantly localized in the cytoplasm of blastoids before Day 3. Nuclear YAP localization was evident in most outside cells in the blastoids at Day 5 (Figure 1B). Similarly, phosphorylated YAP was decreased (i.e., increased nonphosphorylated YAP in TE nuclear localization) at Day 5 compared with Day 3 in blastoids (Supplementary Figures S4A,B), which was consistent with the change in phosphorylated YAP during mouse early embryo development (Supplementary Figure S4C) (Rayon et al., 2014; Posfai et al., 2017). Previous studies found phosphorylation changes in multiple enzymes participating in glycogenesis downstream of insulin signaling (Gao et al., 2017). At the protein level, glycogen synthase 1 (GYS1) was rapidly decreased in the morula stage (Supplementary Figure S4E). Key enzymes in glycogenesis are gradually degraded, and those in glycogenolysis are increased during embryo development. However, in the blastoids, these key enzymes were all increased except for GYS1, which was stable in blastoids from Day 3 to Day 5 (Supplementary Figure S4D). Furthermore, the detected phosphorylated sites of GYS1 in blastoids were different from those in mouse embryos (Supplementary Figures S4F,G). In early preimplantation embryos *in vivo*, the glycogen content is high from MII to the morula stage but degrades during the blastocyst stage (Thomson and Brinster, 1966). These results indicated that abnormal glycogen metabolism may lead to differences between blastoids and mouse embryos and provide clues to improve the generation of mouse blastoids.

## Discussion

In this study, we revealed the protein expression profile of blastoids and metabolite characteristics. Furthermore, the protein phosphorylation sites were identified to show the phosphoproteomic analysis in blastoids compared with mouse early embryos. Clustering analysis revealed that the protein profile of blastoids at Days 3 and 5 was more similar to that of 8-cell to morula stage embryos. As the protein expression profile underwent a major shift in the blastocyst stage, the protein expression in blastoids at Day 5 was distinct from that at the blastocyst stage and more similar to that at the morula stage. This may be a result of blastoid implantation failure and further development to birth, although the RNA profile is more similar to blastocysts for blastoids at Day 5 (Gao et al., 2017). Furthermore, phosphorylation protein analysis revealed abnormal glycogen and glucose metabolism, indicating the contribution to the difference between blastoids and natural mouse embryos. Glucose is necessary for TE marker activation and specification in mouse early embryos. Glucose is transported to the cytoplasm of embryos from the external environment, supporting development through multiple pathways. It controls the nuclear translocation of YAP through the hexosamine biosynthetic pathway (HBP). Then, it mediates mTOR pathway activation to control TFAP2C translation. Nuclear-localized YAP and TFAP2C form a complex to activate CDX2 expression for TE specification (Chi et al., 2020; Zhu and Zernicka-Goetz, 2020). In EPS blastoids, the requirement of glucose for TE-like cell specification and blastoid cavity formation was also shown in the 2-DG treatment experiment (Figure 2F). In recent years, haploid TSCs (haiTSCs) from the trophoblast layer has been established and become a platform to explore the mechanisms of extraembryonic lineage specification and placental development (Peng et al., 2019; Xu et al., 2021). For further exploring the function of glucose for TE-like cell specification, haiTSCs will be a powerful tool. Moreover, haiTSCs could provide cues on how to form normal TE-like cells owing to their single genome and advantages of genetic screening. Above all, our study revealed the protein expression profile of EPS blastoids compared with mouse embryos during preimplantation development and indicated that glucose metabolism is key to blastoid formation.

## Data Availability

The datasets presented in this study can be found in online repositories. The names of the repository/repositories and accession number(s) can be found below: ProteomeXchange Consortium via the PRIDE partner repository with the dataset identifier PXD031002.
