# Supplementary figures and images for: Protein Expression Landscape Defines the Formation Potential of Mouse Blastoids From EPSCs
Source: Front Cell Dev Biol. 2022 Feb 8;10:840492. doi: 10.3389/fcell.2022.840492 (PMC8861521; doi:10.3389/fcell.2022.840492)

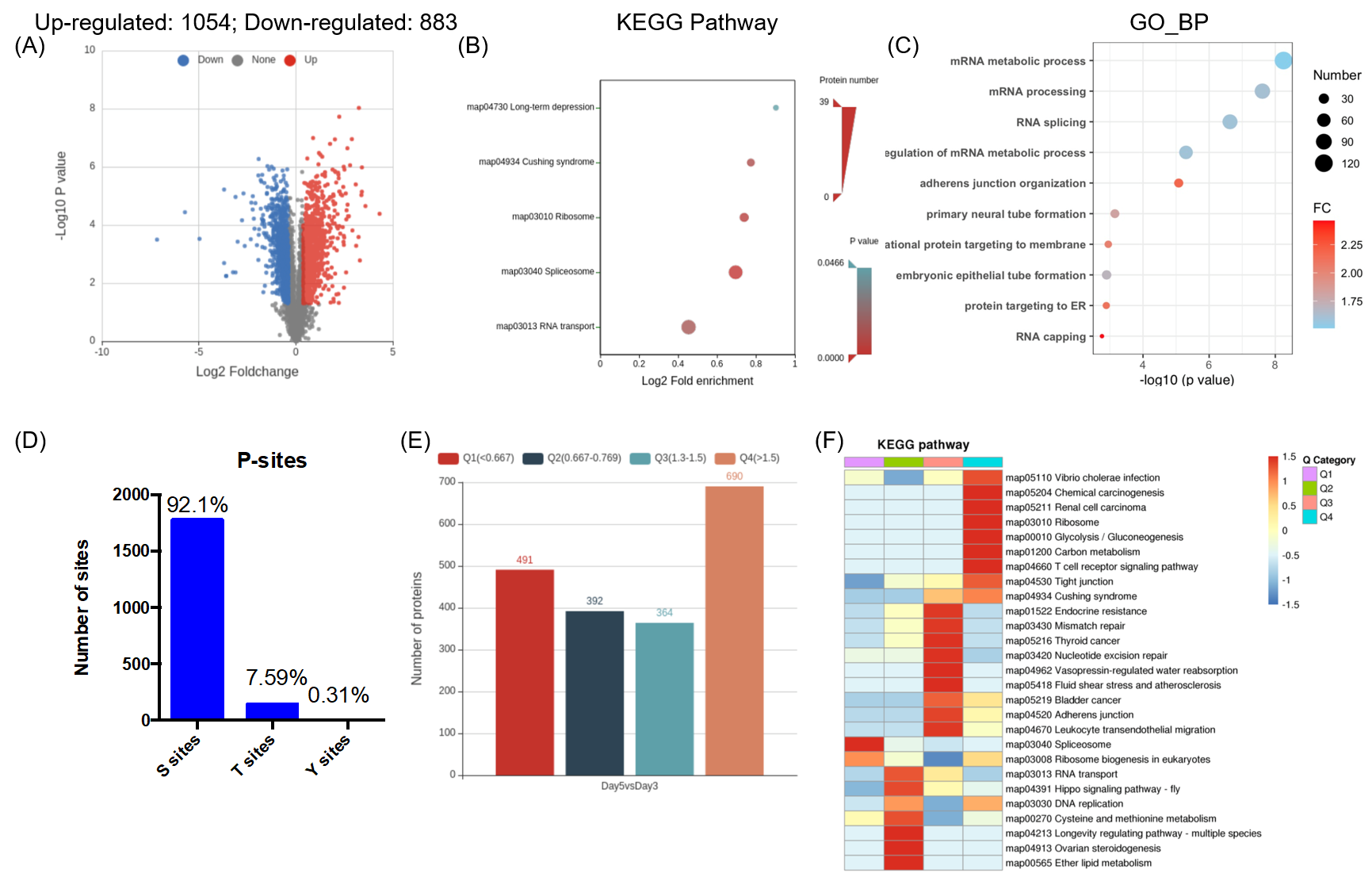

Supplement: Supplementary file 1 [file Image3.TIF]

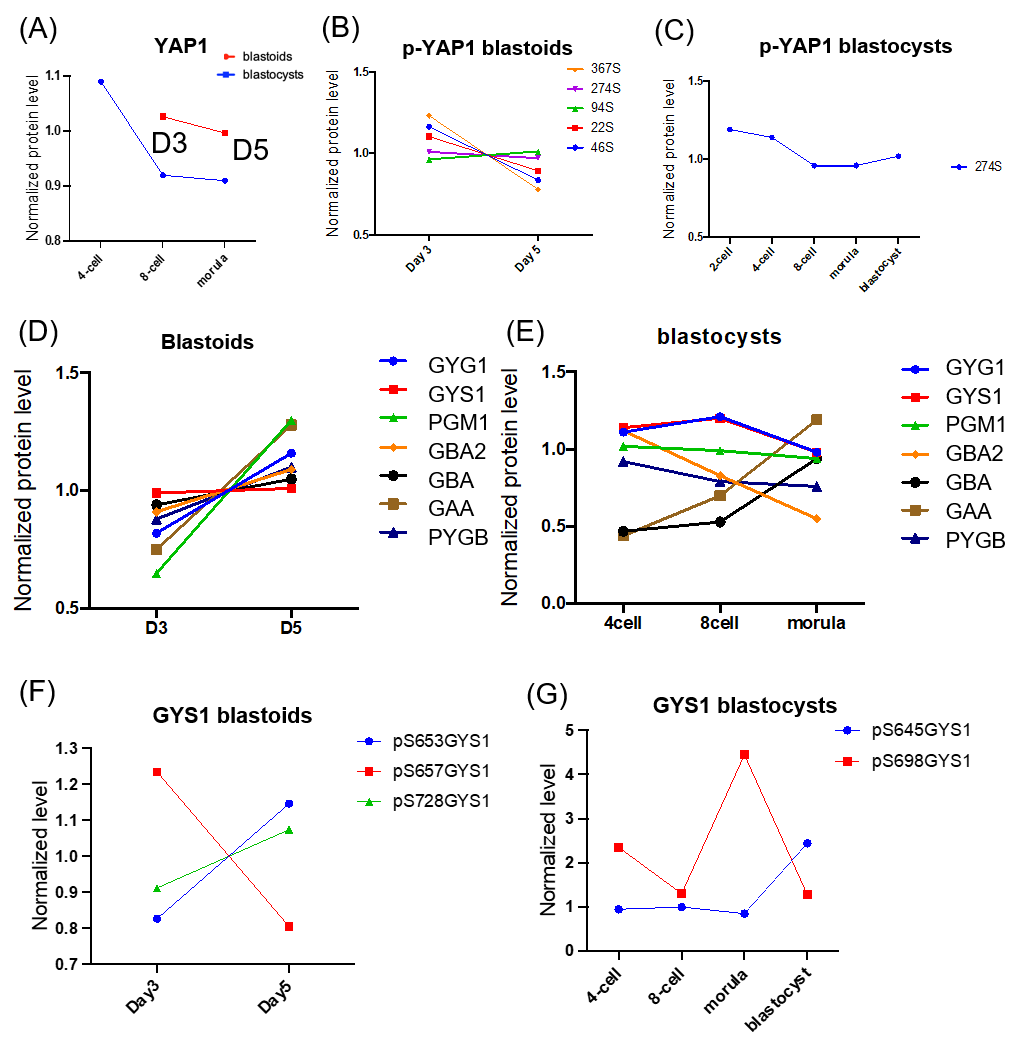

Supplement: Supplementary file 2 [file Image4.TIF]

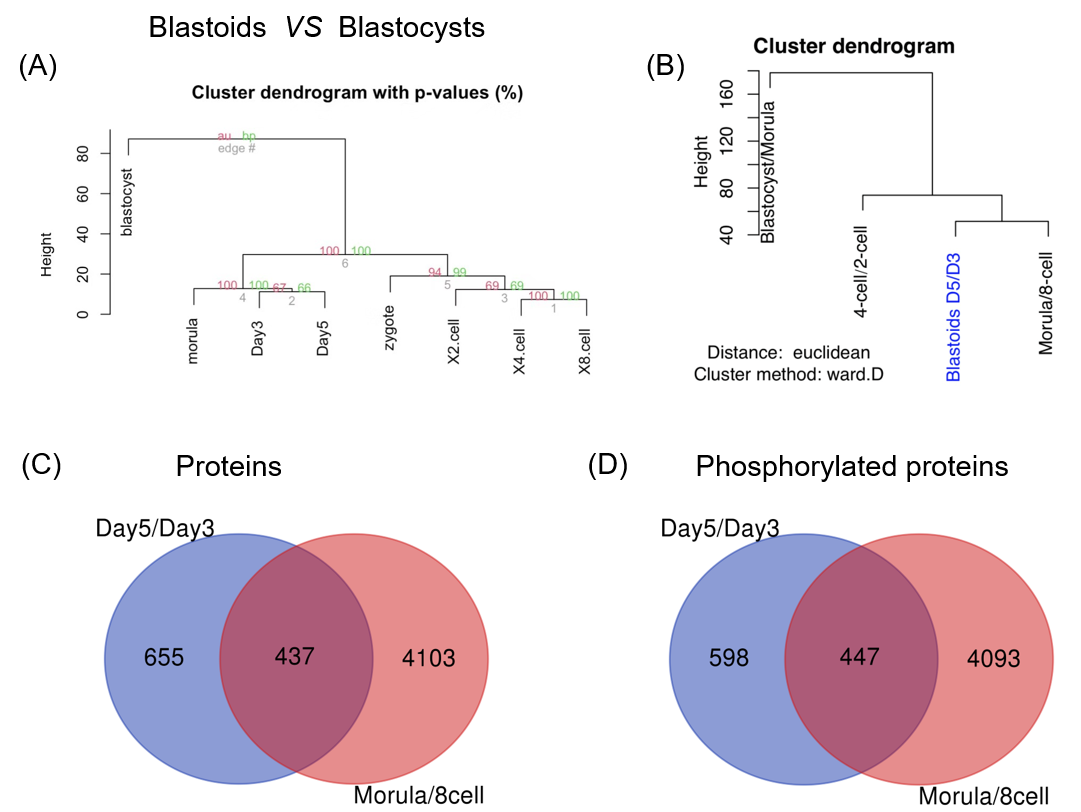

Supplement: Supplementary file 3 [file Image2.TIF]

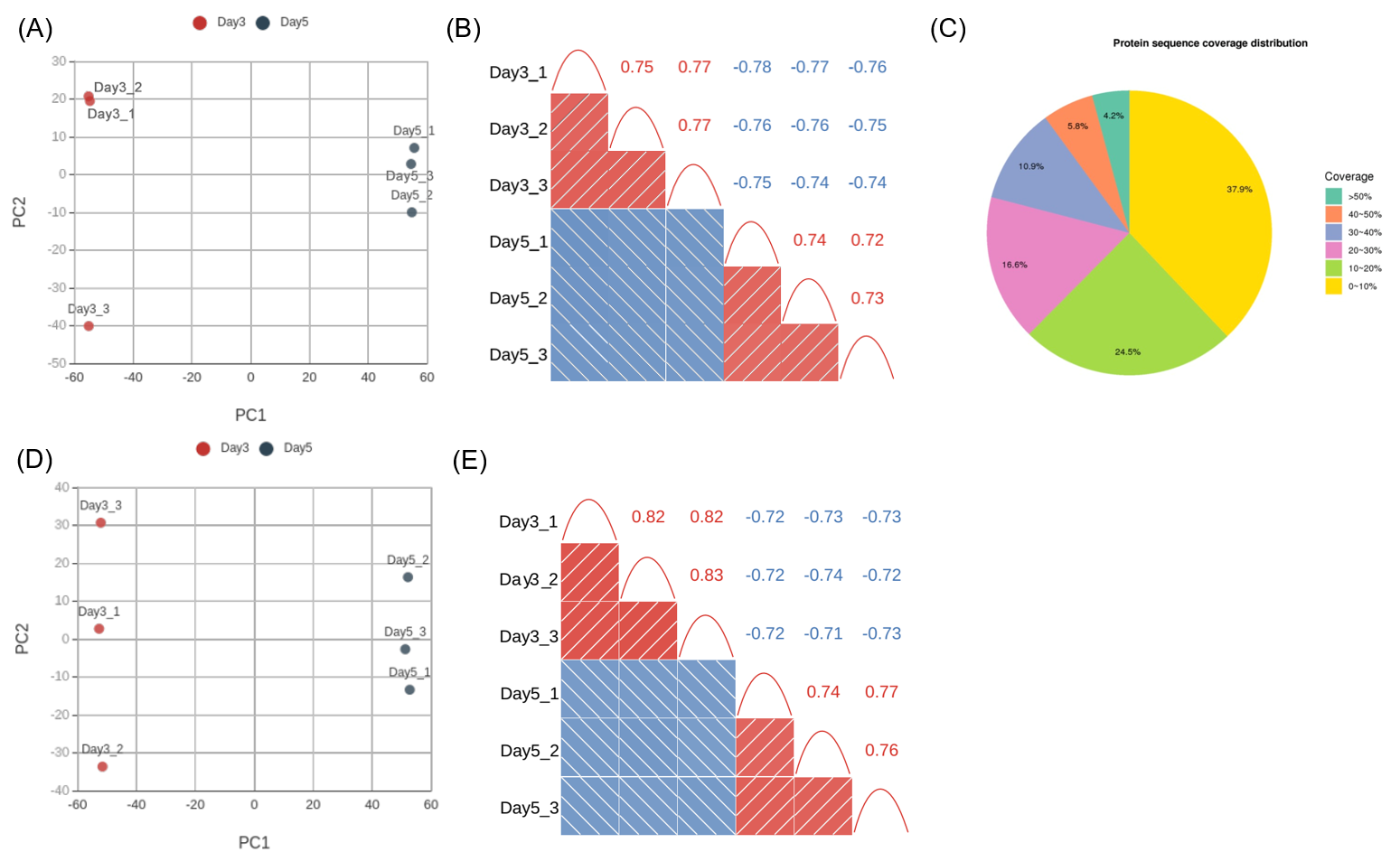

Supplement: Supplementary file 4 [file Image1.TIF]
